# Which classroom anxiety experience should teachers target first? A network-simulation study of foreign language classroom anxiety and enjoyment

**DOI:** 10.3389/fpsyt.2026.1994670

**Published:** 2026-10-13

**Authors:** Xia Yu, Qian Zhou

**Affiliations:** 1 School of Languages and Literatures, Chengdu College of Arts and Sciences, Chengdu, China; 2 School of Foreign Languages, Southwest Jiaotong University, Chengdu, China

**Keywords:** English as a second language (ESL), foreign language classroom anxiety (FLCA), foreign language enjoyment (FLE), network simulation, psychological network analysis

## Abstract

**Background:**

Foreign language classroom anxiety and enjoyment are moderately negatively related, but this relationship has been examined almost entirely at the total score level, and to our knowledge, no published study has asked which specific anxiety experiences are most closely coupled to enjoyment.

**Methods:**

Using item-level psychological network analysis and the GGMNIRA simulated node-manipulation framework, we estimated the joint network of eight anxiety items and nine enjoyment items in 3,957 Chinese non-English-major college students and simulated the alleviation of each anxiety node in turn.

**Results:**

Rank-based stability was high (case-dropping correlation stability coefficient ≥ 0.75 for EI, BEI, and the KL rankings). The highest expected influence nodes were shared jokes and topics and teacher support; among anxiety items, panic without preparation speaking ranked highest. The simulations applied two complementary criteria that answer different questions: panic without preparation produced the largest distributional reconfiguration of the enjoyment construct, whereas alleviating nervousness when speaking predicted the largest signed enjoyment gain, the largest model-implied gain among the eight anxiety items. The highest bridge-centrality anxiety node showed negligible simulated effects.

**Conclusions:**

Speaking-related anxiety, particularly the fear of speaking without preparation, is the anxiety experience most closely coupled to enjoyment. Simulated conditioning offers a principled, model-implied complement to centrality when researchers generate hypotheses about which anxiety experiences classroom practice might target first.

## 1 Introduction

Foreign language classrooms are emotionally charged settings, and among the emotions learners experience there, anxiety and enjoyment have drawn the most sustained research attention over the past four decades (1, 2). In China, college English is compulsory for nearly all non-English majors, and classroom speaking tasks, frequent assessment, and peer comparison keep the emotional stakes high (3). How these two emotions connect at the level of specific classroom experiences, and whether acting on particular anxiety-provoking situations could lift enjoyment, bears directly on how teachers design classroom interaction.

Foreign language classroom anxiety also connects to forms of anxiety that psychiatric research on young adults examines directly. Its defining situations (speaking in front of others, being evaluated, fearing mistakes) overlap with the core fears of social anxiety disorder, one of the most prevalent anxiety conditions in this age group (4). Public-speaking fear is reported by roughly one-third of community samples and is accompanied by significant distress or impairment in a substantial minority (5). In language classrooms, this evaluative anxiety shows up as a measurable reluctance to speak: among Chinese EFL learners, anxiety is a robust predictor of unwillingness to communicate in English (6). Knowing which speaking-related classroom experiences are most tightly coupled to students’ enjoyment may therefore be useful not only for language teaching but also for mental-health work with university students.

Foreign language classroom anxiety refers to the worry and physiological arousal associated with learning and using a second language in class (1). The construct is measured chiefly by the 33-item Foreign Language Classroom Anxiety Scale (FLCAS; 1), and a validated eight-item short form (S-FLCAS) has since been developed and psychometrically examined (7). Enjoyment entered second language acquisition research later, through the positive psychology turn that MacIntyre and Gregersen championed (2). It captures the pleasure and reward learners feel in the classroom and is measured by the Foreign Language Enjoyment Scale and its nine-item short form (S-FLES), which covers teacher appreciation, personal enjoyment, and social enjoyment (8, 9).

Anxiety and enjoyment correlate negatively, but only moderately. The earliest large-sample estimate placed the correlation at *r* = −0.36, with the two emotions sharing only approximately 13% of their variance (9), and a meta-analysis converged on a similar value of *r* = −0.31 (10). Estimates obtained with Chinese university samples range from −0.44 (3) to −0.21 (11), and longitudinal work reports a factor-score correlation of approximately −0.49 (12). Two features of this literature matter for the present study. First, the association is consistently negative but far from perfect: less anxiety does not automatically mean more enjoyment. Second, the two emotions appear to have different sources; enjoyment tracks teacher-related and environmental factors, while anxiety tracks learner-internal and task-related factors (3, 9, 13). This asymmetry suggests a directional question that the correlational literature has not answered: if particular anxiety experiences could be reduced, how much of the enjoyment gain would follow? Anxiety does respond to treatment. A 6-week AI-assisted speaking intervention cut classroom anxiety with a large effect (*d* = −0.81) while enjoyment rose in parallel (*d* = 0.45) (14), and a positive-psychology classroom program reduced anxiety in college language courses (15). Such work, however, changes whole learning environments or whole treatments rather than single anxiety experiences. Teachers who must use their limited classroom time wisely need to know which experiences, among the many that make learners anxious, are most tightly coupled to enjoyment. That requires moving from the aggregate to the item and from description to simulated intervention.

Two further developments motivate the present approach. The first is measurement granularity. Nearly all prior studies operate at the total score level, yet the two scales are heterogeneous collections of concrete classroom situations, such as *fear of speaking without preparation*, *worry about mistakes*, and *shared laughter in class*. Item-level information is lost when scores are aggregated. Total scores can also mask item-level differences: gender differences in anxiety and enjoyment appear on a handful of items, while total scores show none (16). Which items do the linking work between the two constructs is exactly the kind of question aggregation cannot answer. The second development is network psychometrics. Psychological networks treat symptoms or items as nodes connected by conditional dependence relations, and their accuracy and stability can be formally evaluated (17, 18). Network analyses have recently entered second language emotion research. A cross-lagged panel network showed that achievement predicts emotions more strongly than the reverse among Chinese middle-school learners (19). A dimensional network of anxiety, boredom, and grit in Chinese college learners left enjoyment out deliberately and called for networks containing both positive and negative classroom experiences (20). An item-level network of the full FLCAS identified general tenseness as the most central item (21). In these networks, nodes are items (or item clusters) and edges are regularized partial correlations, so a connection between two nodes indicates that they co-occur beyond what the other nodes already explain.

Network studies published so far, however, share a limitation: they select “important” nodes through centrality indices, which describe structural connectedness, not causal influence (17, 22). Authors in applied linguistics have begun to note this gap, observing that centrality does not establish that a node causally activates other experiences (20). To our knowledge, no published study has asked what happens to enjoyment when a specific anxiety node is manipulated in simulation. Simulation offers a principled way forward: the GGMNIRA framework recently formalized how to manipulate the conditional mean of a node in a Gaussian graphical model and how to quantify the resulting change in a target construct through Kullback–Leibler divergence, with stability and difference-testing procedures to support the rankings (23).

The present study therefore constructed an item-level joint network of the eight S-FLCAS items and the nine S-FLES items among 3,957 Chinese non-English-major college students and applied GGMNIRA to simulate manipulations of each anxiety node. We address three research questions: (1) What is the structure of the joint anxiety–enjoyment network, and which items are most central in terms of standardized expected influence (EI) and bridge expected influence (BEI)? (2) Which anxiety node, when its conditional mean is shifted in simulation, produces the largest distributional reconfiguration of the enjoyment construct (a direction-agnostic, profile-level criterion indexed by Kullback–Leibler divergence)? (3) Does simulated alleviation of anxiety nodes predict higher enjoyment, and if so, which node yields the largest predicted gain per one-standard-deviation reduction of the anxiety node’s latent conditional mean? Answers to these questions move the field from describing the anxiety–enjoyment correlation toward identifying the specific anxiety experiences that are most tightly coupled to enjoyment in model-implied prediction. In short, we ask not whether anxiety and enjoyment hang together, but which anxiety experiences are most closely coupled to enjoyment at the item level.

## 2 Methods

### 2.1 Participants and procedure

Participants were recruited in May 2024 from a single public university in Sichuan Province, China, whose national ranking approximates the median of Chinese universities. The survey primarily targeted first-year students enrolled in the compulsory first-year college English course; students from other grades were surveyed on a supplementary basis. All instruments were administered in Chinese via the Wenjuanxing online survey platform, which required a response to every item before submission, and the analyzed dataset therefore contained no missing values. The survey was not distributed or invigilated by students’ own English instructors. Of the 5,120 students invited, 4,387 completed the questionnaire (response rate = 85.7%). After excluding 430 English majors, the final sample comprised 3,957 non-English-major undergraduates (2,416 female students, 61.1%; 1,541 male students, 38.9%). Most were first-year students (*n* = 3,374, 85.3%), followed by third-year students (*n* = 469, 11.9%), with the remainder (*n* = 114, 2.9%) distributed across the other grades. Age was collected in categories: 19 years (*n* = 1,858, 47.0%), ≤18 years (*n* = 857, 21.7%), 20 years (*n* = 576, 14.6%), ≥22 years (*n* = 420, 10.6%), and 21 years (*n* = 246, 6.2%). Han students accounted for 90.6% of the sample, and 70.9% reported a rural household registration. All students provided informed consent, and the study was approved by the Academic Ethics Committee of Chengdu College of Arts and Sciences (No. 20240232017) and conformed to the Declaration of Helsinki.

### 2.2 Measures

#### 2.2.1 Foreign language classroom anxiety

Anxiety was measured with the eight-item short form of the Foreign Language Classroom Anxiety Scale (S-FLCAS; 4), which was originally selected from the 33-item FLCAS, whose psychometric foundations were established with college learners (1, 24); the eight-item short form has also been validated in Chinese university students with factor-analytic and item-response-theory procedures (25). Items (e.g., “I start to panic when I have to speak without preparation in language class”) were rated on a 5-point Likert scale from 1 (strongly disagree) to 5 (strongly agree). One item (ANX5, “I feel confident speaking English in class”) is reverse-worded; it was recoded before analysis (recoded score = 6 − raw score) so that higher scores indicate greater anxiety throughout. The S-FLCAS has shown good reliability in Chinese university samples (14); in the present sample, Cronbach’s *α* was 0.93.

#### 2.2.2 Foreign language enjoyment

Enjoyment was measured with the nine-item short form of the Foreign Language Enjoyment Scale (S-FLES; 5), rated on the same 5-point scale. The instrument comprises three subscales of three items each: teacher appreciation (e.g., “My English teacher is supportive”), personal enjoyment (e.g., “I enjoy learning English”), and social enjoyment (e.g., “We laugh a lot in my English class”). The parent enjoyment scale has a validated Chinese version (26), and the S-FLES has shown good reliability with Chinese university students (27). In the present sample, Cronbach’s *α* was 0.97.

### 2.3 Data analysis

#### 2.3.1 Gaussian graphical model

All 17 items formed the nodes of a Gaussian graphical model estimated with the graphical LASSO together with the extended Bayesian information criterion, a regularized approach that selects an interpretable sparse network by shrinking weak edges toward zero (28). Because the items were ordinal, the model used polychoric correlations (automatic correlation selection), and the EBIC tuning parameter was set to the conventional value of 0.5. Expected influence and bridge expected influence were computed for every node and standardized before ranking. Edge-weight accuracy was examined with 5,000 non-parametric bootstrap samples and 95% confidence intervals, and the stability of the centrality indices was assessed with case-dropping subset bootstrap and reported as the correlation stability coefficient, with values above 0.25 regarded as acceptable and above 0.5 as good (18). Network density and community structure were examined descriptively, and the two-construct (anxiety/enjoyment) partition assumed by the bridge indices was checked against data-driven community detection (exploratory graph analysis and walktrap clustering; **Supplementary Section SA8**). Because the three teacher-appreciation enjoyment items proved to be near-duplicates, topological overlap was assessed with unique variable analysis (29) and the goldbricker procedure, and all main analyses were re-run with those items combined into a single composite node as a sensitivity analysis (**Supplementary Section SA7**).

#### 2.3.2 GGMNIRA simulated node manipulation

The novel element of this study is the simulation step, which follows the GGMNIRA framework (23). In a standardized Gaussian graphical model, the expected value of every node is a weighted combination of its neighbors’ values. GGMNIRA therefore simulates conditioning on a shifted conditional mean: a chosen amount *δ*, expressed in marginal standard-deviation units of the standardized (polychoric) metric, is added to the node’s conditional-mean equation, *μ′i|\i* = *Σj*≠*i βˆji Xj + δ*, where the *βˆji* are the regression coefficients implied by the regularized EBICglasso precision matrix (the matrix *B* with entries *−Θij/Θii*). Because the shift propagates through the whole network, the equilibrium response of all nodes is *δ* times the corresponding column of the propagation matrix *M* = (*I* − *B*)^-^¹. The reported effects are therefore marginal, model-implied predictions in which all other nodes, including the other anxiety nodes, are allowed to co-vary, rather than partial effects computed with the remaining network held fixed. This marginal estimand matches the classroom scenarios discussed below, in which alleviating one anxiety experience would naturally co-occur with change in the experiences connected to it; a partial, direct path view of each node’s association with enjoyment is provided by the regularized partial-correlation edge weights reported in **Supplementary Table S3**. Since the variables are standardized, *δ* is in marginal rather than node-specific conditional *SD* units (*1*/√*Θjj*); because conditional *SD*s differ across nodes, the same *δ* corresponds to different numbers of conditional *SD*s per node, a property we note explicitly here. The effect of the simulated conditioning is quantified by the Kullback–Leibler divergence between the distribution of the outcome construct before and after the shift: the larger the divergence, the more strongly the conditioned node reshapes the outcome distribution. Because only the eight anxiety nodes were manipulated, and the enjoyment construct served as the outcome, all effects were evaluated on the marginal distribution of the enjoyment construct (i.e., on its marginal precision matrix), as the framework prescribes. First, we simulated, for each of the eight anxiety nodes in turn, a shift of *δ* = 1 and recorded the resulting KL divergence (in nats) of the enjoyment marginal distribution; because KL is a quadratic form in *δ*, this criterion is direction-agnostic and indexes how strongly a node reshapes the profile of the enjoyment distribution rather than how much enjoyment it adds. (The framework’s chi-square-based effectiveness criterion and the minimum manipulation strength derived from it are monotone transformations of the KL value and are reported only in the **Supplementary Table RA13**) Second, we simulated alleviation of each anxiety node (*δ* = −1, i.e., a reduction of one marginal latent standard deviation applied to the node’s conditional mean; predicted changes scale linearly in |*δ*|) and recorded the predicted change in the sum of the nine enjoyment latent variables, a signed, direction-aware criterion expressed as the sum of nine per-item shifts, each in marginal *SD* units of the standardized metric (neither in raw 1–5 score points nor relative to the composite’s *SD*). Third, as transparency benchmarks, we regressed the enjoyment composite on the eight anxiety items (**Supplementary Table RA10**) and ran the reverse-direction simulation, conditioning each enjoyment node and predicting anxiety (**Supplementary Table RA11**). The GGMNIRA framework also supplies stability and inference procedures of its own: a case-dropping stability coefficient for the KL rankings (KL-CS) and non-parametric bootstrap pairwise difference tests among node effects. The pairwise difference tests were run at a single common manipulation strength (*δ* = 3) so that all pairwise differences share one scale. Because KL values, and hence their pairwise differences, scale multiplicatively with *δ*², the identity of the significant pairs does not depend on the chosen intensity; it serves only as a common computational yardstick. The tests cover the 28 unique (unordered) node pairs, and no multiplicity correction was applied, following the framework’s current recommendation. Approximately 1.4 false positives would be expected at *α* = 0.05 if all null hypotheses were true, and no substantive conclusion rests on any single pairwise test. For the alleviation effects, we additionally computed non-parametric bootstrap 95% confidence intervals and pairwise difference tests (500 accepted resamples, with networks re-estimated under the same polychoric/EBICglasso pipeline as the main analysis; resamples yielding non-positive-definite polychoric matrices were redrawn; **Supplementary Section SA4**). For the KL-based stability coefficient, we followed the benchmarks proposed for GGMNIRA itself (values below 0.25 are not interpretable; values above 0.40 are desirable) (23), which differ from the 0.25/0.50 conventions used for traditional centrality indices.

A complete technical account, including the underlying equations, is available in the GGMNIRA preprint (23). Analyses were conducted in R (30) using the packages qgraph, bootnet, networktools, EGAnet, and GGMNIRA. The de-identified data and the complete analysis code are provided in the online **Supplementary Materials**.

## 3 Results

### 3.1 Descriptive statistics

**Table 1** reports the means, standard deviations, skewness, and kurtosis of the 17 nodes. Skewness ranged from −1.04 to 0.23 and kurtosis from −1.14 to 1.17, and the polychoric correlation matrix was positive definite, so no distribution precluded the polychoric Gaussian graphical model. At the total score level, the model-implied correlation between the unit-weighted anxiety and enjoyment composites (computed from the polychoric matrix) was *r* = −0.16; the observed Pearson correlation between the raw total scores was −0.09 (Spearman −0.24), attenuated by the ordinal skew of the items and by the anomalous positive manifold of the reverse-worded anxiety item (excluding that item: Pearson −0.12, model-implied −0.20).

**Table 1:** Means, standard deviations, skewness, and kurtosis of the 17 items.

Node	Label	*M*	*SD*	Skewness	Kurtosis
ANX1	Fear of mistakes	2.54	1.18	0.12	−1.08
ANX2	Panic without preparation	2.52	1.19	0.11	−1.14
ANX3	Reluctant to volunteer	2.62	1.20	0.02	−1.11
ANX4	Anxious despite preparation	2.33	1.06	0.23	−0.80
ANX5	Low speaking confidence	2.56	1.08	0.09	−0.68
ANX6	Heart pounds when called	2.44	1.12	0.16	−0.95
ANX7	Others better at English	2.46	1.10	0.12	−0.89
ANX8	Nervous when speaking	2.38	1.07	0.21	−0.81
ENJ1	Teacher encourages us	3.76	0.98	−1.00	1.14
ENJ2	Teacher is kind	3.79	0.99	−1.04	1.17
ENJ3	Teacher is supportive	3.79	0.98	−1.03	1.17
ENJ4	Enjoy learning English	3.59	1.03	−0.68	0.29
ENJ5	Learn interesting things	3.64	1.01	−0.81	0.61
ENJ6	Proud of my English	3.37	1.08	−0.40	−0.30
ENJ7	Close study group	3.42	1.07	−0.46	−0.17
ENJ8	Laughter in class	3.44	1.06	−0.50	−0.07
ENJ9	Shared jokes and topics	3.48	1.04	−0.54	0.07

### 3.2 Network structure and centrality

**Figure 1** displays the estimated network. The square orange shapes represent enjoyment nodes, and the circular blue shapes represent anxiety nodes. The strongest edges lay within constructs, whereas cross-construct edges were generally small, the largest connecting *low speaking confidence* (ANX5) with *proud of my English* (ENJ6, *w* = 0.16). **Figure 2** plots the standardized EI and BEI. The two highest EI values were *shared jokes and topics* (ENJ9, *z* = 1.50) and *teacher is supportive* (ENJ3, *z* = 1.15); the highest anxiety-side EI was *panic without preparation* (ANX2, *z* = 1.08). For BEI, the leading anxiety node was *low speaking confidence* (ANX5, *z* = 3.30), and the leading enjoyment node was *learning interesting things* (ENJ5, *z* = 0.43). Stability was adequate: the case-dropping correlation stability coefficients were 0.75 for both EI and BEI (**Supplementary Figure S1**). **Supplementary Figure S2** represents the bootstrapped 95% CIs for all edge weights and suggests that the accuracy is acceptable. The full edge-weight matrix appears in Online **Supplementary Table S3**. The network was moderately dense: 83 of 136 possible edges were non-zero (49 within-construct and 34 cross-construct). Bootstrap centrality difference tests (**Supplementary Section SA8**; **Supplementary Figure S5**) showed that the EI of ENJ9 exceeded that of 12 of the other 16 nodes and the EI of ENJ3 exceeded 9 of 16, which supports describing them as the highest-influence nodes. Data-driven community detection (exploratory graph analysis and walktrap) recovered exactly the anxiety block and the enjoyment block, the latter splitting into the teacher-appreciation triplet (ENJ1–ENJ3) and the remaining six items. No node was misassigned across constructs, which supports the two-construct specification of the bridge indices (**Supplementary Section SA8**).

**Figure 1:**
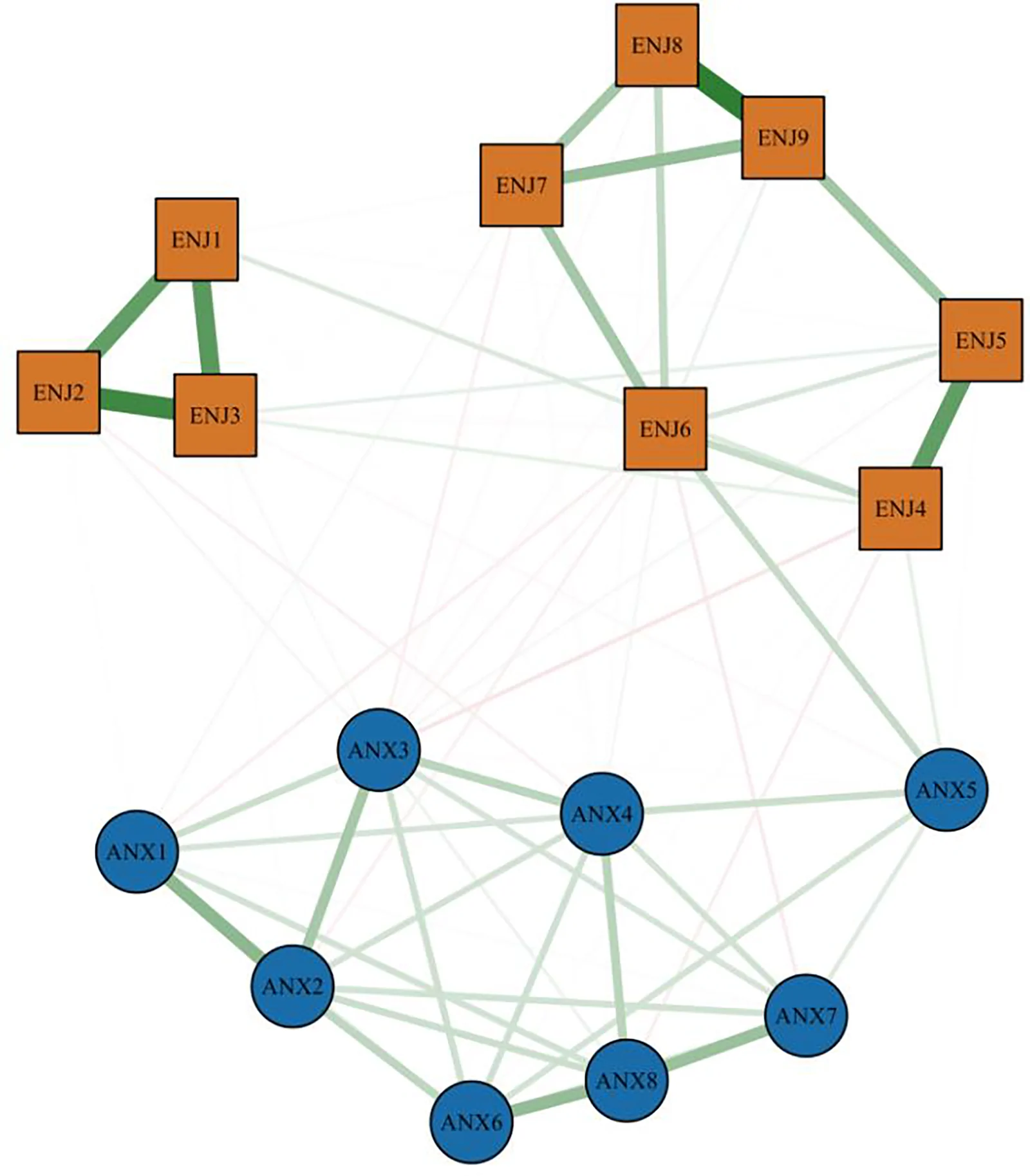
Estimated joint network of foreign language classroom anxiety and enjoyment items. ANX1, fear of mistakes; ANX2, panic without preparation; ANX3, reluctant to volunteer; ANX4, anxious despite preparation; ANX5, low speaking confidence; ANX6, heart pounds when called; ANX7, others better at English; ANX8, nervous when speaking; ENJ1, teacher encourages us; ENJ2, teacher is kind; ENJ3, teacher is supportive; ENJ4, enjoy learning English; ENJ5, learn interesting things; ENJ6, proud of my English; ENJ7, close study group; ENJ8, laughter in class; ENJ9, shared jokes and topics.

**Figure 2:**
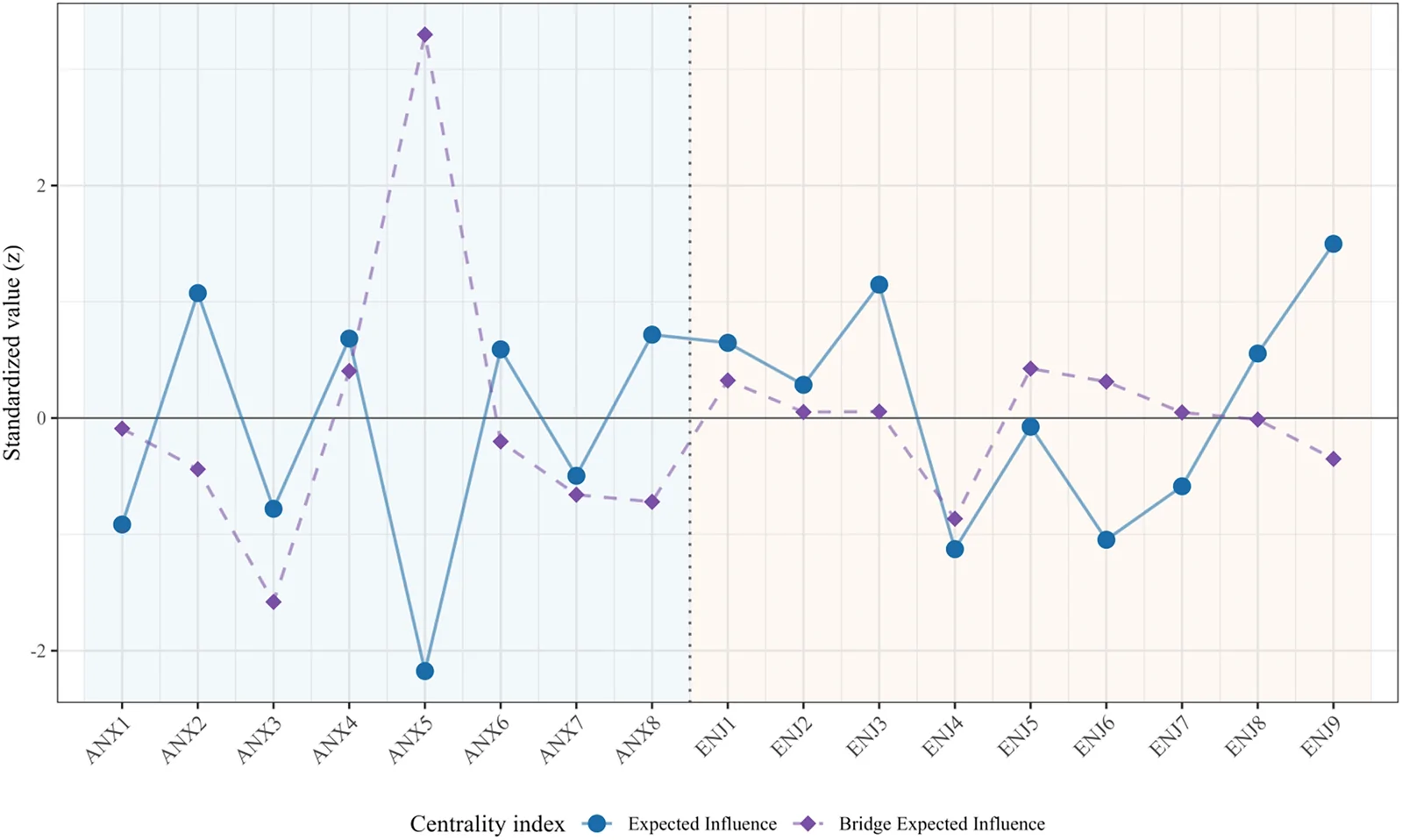
Standardized expected influence and bridge expected influence of the 17 network nodes. ANX1, fear of mistakes; ANX2, panic without preparation; ANX3, reluctant to volunteer; ANX4, anxious despite preparation; ANX5, low speaking confidence; ANX6, heart pounds when called; ANX7, others better at English; ANX8, nervous when speaking; ENJ1, teacher encourages us; ENJ2, teacher is kind; ENJ3, teacher is supportive; ENJ4, enjoy learning English; ENJ5, learn interesting things; ENJ6, proud of my English; ENJ7, close study group; ENJ8, laughter in class; ENJ9, shared jokes and topics.

### 3.3 GGMNIRA simulated manipulation of anxiety nodes

**Table 2** presents, for each anxiety node, the Kullback–Leibler divergence (in nats) of the enjoyment marginal distribution produced by a unit shift of the node’s conditional mean (*δ* = 1; one marginal latent standard deviation). Because KL is a quadratic form in the propagated shift, it is direction-agnostic: it indexes how strongly a node reshapes the profile of the enjoyment distribution, not how much enjoyment it adds. Shifting panic without preparation (ANX2) produced the largest distributional reconfiguration (KL = 1.68), followed by nervousness when speaking (ANX8, KL = 1.55) and reluctance to volunteer (ANX3, KL = 1.23); low speaking confidence (ANX5) had a negligible effect (KL = 0.03). To make the geometry of this criterion explicit, we decomposed each node’s propagated shift into its signed component along the first principal direction of the enjoyment covariance matrix (85.1% of the variance, a general enjoyment factor) and the orthogonal, profile component (**Supplementary Table RA1**). All eight shifts lie predominantly along the first principal direction (74%–99% of the squared shift), and the KL ranking tracks the profile component (*r* = 0.92) rather than the main direction component (*r* = 0.84).

**Table 2:** Simulated effects of a unit shift (*δ* = 1, one marginal latent standard deviation) of each anxiety node’s conditional mean on the marginal distribution of the enjoyment construct, measured by Kullback–Leibler divergence (in nats).

Rank	Node	Label	KL (*δ* = 1)
1	ANX2	Panic without preparation	1.68
2	ANX8	Nervous when speaking	1.55
3	ANX3	Reluctant to volunteer	1.23
4	ANX6	Heart pounds when called	0.95
5	ANX7	Others better at English	0.69
6	ANX4	Anxious despite preparation	0.63
7	ANX1	Fear of mistakes	0.58
8	ANX5	Low speaking confidence	0.03

The alleviation analysis shown in **Figure 3** instead asks a plainer, practice-facing question: how much does the sum of the nine enjoyment latent variables change when a given anxiety node’s latent conditional mean is reduced by one standard deviation (*δ* = −1)? Alleviating nervousness when speaking (ANX8) predicted the largest enjoyment gain (+9.38 summed *SD* units), followed by panic without preparation (ANX2, + 7.26) and anxiety despite preparation (ANX4, + 6.65); all nodes predicted gains except low speaking confidence (ANX5, −1.93). The two criteria answer different questions, and their top nodes differ for an interpretable reason. KL weights the propagated shift by the inverse of the enjoyment covariance matrix (a Mahalanobis-type metric), so shifts in directions that are unusual given how the enjoyment items co-vary contribute most, whereas the alleviation criterion sums the signed per-item shifts with equal weights. ANX2 tops the KL ranking because its propagated shift has the largest profile-reshaping component; ANX8 tops the alleviation ranking because its shift aligns most strongly with the main enjoyment direction and sums to the largest total gain. The two rankings agree in placing speaking-related anxieties at the top, but they differ in which speaking node they highlight, most sharply for anxiety despite preparation (ANX4), which ranks sixth in KL but third in signed gain.

**Figure 3:**
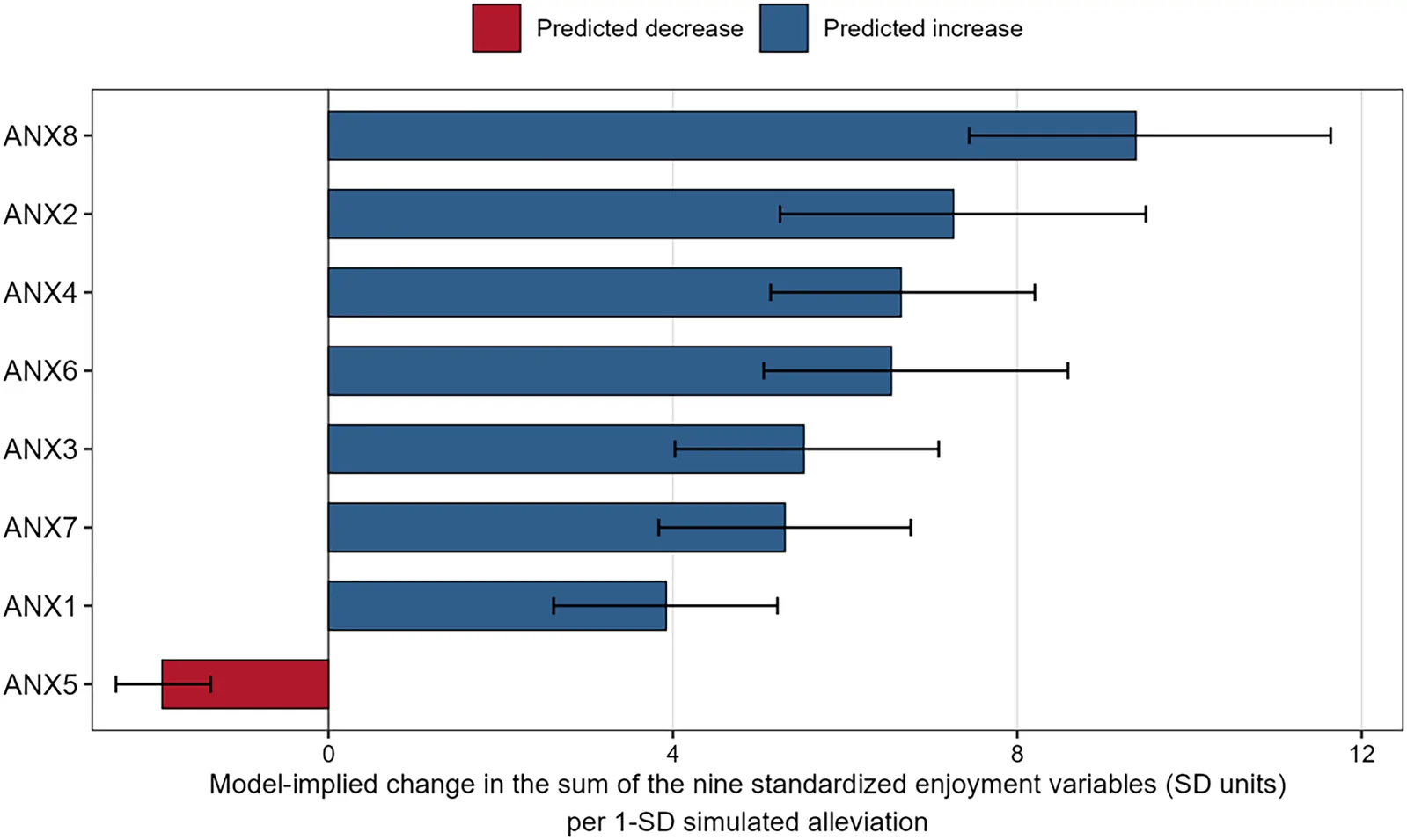
Model-implied change in the sum of the nine enjoyment latent variables (each standardized; *SD* units) per 1 − *SD* simulated alleviation (*δ* = −1) of each anxiety node’s latent conditional mean. Error bars are 95% non-parametric bootstrap confidence intervals. ANX1, fear of mistakes; ANX2, panic without preparation; ANX3, reluctant to volunteer; ANX4, anxious despite preparation; ANX5, low speaking confidence; ANX6, heart pounds when called; ANX7, others better at English; ANX8, nervous when speaking.

Stability and inference for the simulation results were satisfactory. The case-dropping stability coefficient of the KL rankings was 0.75 (benchmark 0.40; 0.75 is the maximum proportion tested). Non-parametric bootstrap difference tests on the KL values showed 21 of the 28 unique pairwise comparisons significant: the top tier (ANX2, ANX8, ANX3) did not differ among themselves, each exceeded the middle tier (ANX1, ANX4, ANX6, ANX7), and ANX5 fell below all seven remaining nodes. For the alleviation effects, bootstrap 95% confidence intervals excluded zero for all eight nodes (**Figure 3**), and pairwise difference tests showed 24 of 28 comparisons significant. Under this signed criterion, ANX8 exceeded both ANX2 and ANX3 significantly, so the two criteria rank the top tier differently rather than merely noisily. The difference-test figure and tables appear in the **Supplementary Materials** (**Supplementary Figure S4**) and **Supplementary Analyses** (**Supplementary Section SA4**), respectively.

Four sensitivity and benchmark analyses, reported in full in the **Supplementary Analyses**, qualify these results. Re-running the network and both simulations without ANX5 left every ranking essentially unchanged (KL: Spearman *ρ* = 1.00; alleviation: *ρ* = 0.96; Section SA6). Combining the near-duplicate teacher-appreciation items (ENJ1–ENJ3) into a single composite node preserved both rankings (*ρ* = 0.98) and the identity of every top node, while attenuating the largest alleviation effect from +9.38 to +7.70, a direct quantification of how much the redundant triplet contributes to the headline magnitude (Section SA7). A multiple regression of the enjoyment composite on the eight anxiety items (*R*² = 0.078) produced a ranking uncorrelated with the alleviation ranking (*ρ* = −0.02) and was dominated by the ANX5 artifact, which underscores that the simulation’s contribution lies in item-level profiles, distributional information, and propagated indirect paths rather than in conditional slopes toward a composite (**Supplementary Table RA10**). The reverse-direction simulation was not a mirror image of the forward direction, being dominated by the high-precision teacher-appreciation items (**Supplementary Table RA11**). All simulated effects are also re-expressed on the original 1–5 response scale in **Supplementary Table RA12**, together with an explicit caution that they are linear extrapolations: the manipulation strengths at which effects become “effective” exceed the observed range of the items, so the rankings, not the absolute magnitudes, carry the interpretation.

## 4 Discussion

### 4.1 Item-level coupling of anxiety and enjoyment

To our knowledge, this is the first study to combine item-level network analysis with simulated node manipulation to examine the anxiety–enjoyment relationship in second language learning. We estimated the joint network of eight anxiety items and nine enjoyment items in 3,957 Chinese non-English-major college students, ranked nodes by expected influence and bridge expected influence, and then simulated manipulations of each anxiety node with GGMNIRA (23). Three findings emerged. First, the strongest expected influence nodes were social and teacher-related enjoyment items, whereas the highest anxiety-side influence belonged to panic about unprepared speaking. Second, the highest bridge node on the anxiety side (low speaking confidence) turned out to be almost irrelevant as a simulated target once conditioning was applied, a discrepancy that shows why centrality alone can mislead. Third, simulated alleviation of speaking-related anxiety predicted the largest enjoyment gains, which supports the proposition that reducing specific anxiety experiences may help brighten classroom enjoyment and gives teachers a ranked, model-generated set of hypotheses rather than a global prescription.

The expected influence findings sit comfortably within the broader literature. Teacher appreciation and classroom atmosphere have repeatedly emerged as the strongest sources of foreign language enjoyment (3, 9, 11), and our two leading nodes, teacher support and shared jokes and topics, belong to that tradition while extending it to the item level. They also refine it: the most influential enjoyment experiences are social, which fits classroom-environment accounts in which cohesion and teacher warmth drive enjoyment far more than individual ability does (13). The prominence of panic about unprepared speaking among anxiety items matches the centrality of speaking-related experiences reported for the full 33-item FLCAS (21) and the long-standing observation that speaking is the skill most strongly associated with language anxiety (1). That the most influential anxiety item concerns unprepared speaking is itself instructive: the perceived lack of readiness, not speaking as such, appears to be the psychological core of the experience.

### 4.2 Why structural prominence and simulated consequence diverge

The bridge-centrality results call for a more cautious reading, and their contrast with the simulation results is the methodological lesson of this study. Low speaking confidence (ANX5) carried the highest standardized BEI among anxiety nodes, which in the traditional centrality logic would single it out as the bridge between anxiety and enjoyment. Yet, its simulated effects were negligible, and it was the only node whose alleviation predicted a decline in enjoyment. On the enjoyment side, learning interesting things (ENJ5) was the leading bridge node, which is consistent with qualitative evidence that interesting content is a core source of enjoyment (31, 32) and with network evidence that interest-related experiences also touch anxiety (19). More decisive is the divergence between ANX5’s bridge prominence and its null simulated effect: it shows concretely that high connectedness does not imply high simulated consequence, the very point emphasized in the network literature (17) and now acknowledged within applied linguistics (20). This study answers that critique with a method rather than a claim: instead of treating centrality as a proxy for a causal target, we simulated the manipulation itself and ranked nodes by its consequences.

The KL divergence is a precision-weighted, distribution-level criterion: it weights the propagated shift by the inverse of the enjoyment items’ covariance matrix (a Mahalanobis-type metric), so shifts in directions that are unusual given how the enjoyment items co-vary contribute more. It is direction-agnostic and says nothing about whether enjoyment rises or falls. The alleviation criterion instead uses an equal-weight, signed composite (the sum of the predicted changes of the nine standardized enjoyment variables) and therefore ranks nodes by predicted enjoyment gain. Panic without preparation (ANX2) tops the first criterion and nervousness when speaking (ANX8) the second. In the classroom, both answers still point the same way: speaking-related anxiety, and especially the fear of speaking without preparation, is the anxiety experience most tightly coupled to enjoyment.

### 4.3 Implications for classroom practice and student wellbeing

The analyses suggest a coherent classroom story, and they generate concrete, testable hypotheses for instructional design rather than prescriptions. The moves suggested by the identified items cost little: give students preparation time and scaffolding before speaking tasks so that “unprepared” no longer applies; normalize improvised speech through low-stakes, ungraded talk so that being put on the spot loses its threat; and sequence speaking activities so that anxiety-triggering situations are defused before enjoyment is asked to appear. Under control-value theory, such changes target the negative activating emotion of anxiety in an outcome-focused situation and, by releasing activity-focused positive emotion, allow enjoyment to do its broadening and resource-building work (33, 34). We stress that these suggestions are derived from item wording and model-implied predictions, not from intervention evidence; the field experiments proposed below would be needed to test them. For student mental health more broadly, the results add item-level detail to a familiar picture: evaluative, speaking-focused classroom situations are the anxiety experiences most tightly coupled to how much students enjoy their learning, and they are also the situations most amenable to low-stakes, supportive classroom design. We emphasize that both instruments are subclinical self-report measures of classroom emotions, and no diagnostic inference about anxiety disorders is intended or licensed by these data. Our results also refine the correlational literature: because anxiety and enjoyment share only part of their variance (9, 10), not every anxiety node is equally coupled to enjoyment, and the simulation makes that coupling explicit node by node. The near-zero role of low speaking confidence (ANX5) additionally cautions against treating a scale’s reverse-worded items as interchangeable with the rest, a practical measurement note for future network studies. Methodologically, the study shows that hypotheses about intervention targets can be generated from simulated consequence, not from structural prominence: the ranking produced by manipulation diverged from the ranking produced by centrality, and the manipulation ranking survived its own stability and difference-testing checks.

### 4.4 Limitations and future directions

Several limitations bound these conclusions. First, the data are cross-sectional and the network is undirected, so the simulated manipulations are model-implied predictions (simulated conditioning) rather than effects of an actual intervention, and no causal inference should be read into the rankings; cross-sectional network quantities are not substitutes for causal identification (22). Second, the manipulation strengths are expressed in standard-deviation units that may correspond to substantial real-world changes, and the simulated effects are linear extrapolations: the strengths at which effects become appreciable exceed the observed range of the items, so only the rankings, not the absolute magnitudes, should be interpreted. Third, language proficiency or achievement was not measured, although it is a plausible common cause of both anxiety and enjoyment, particularly of self-evaluative items such as ENJ6; longitudinal evidence indicates that achievement predicts subsequent emotions at least as strongly as the reverse (19, 35), so part of the conditional anxiety–enjoyment dependence we report may reflect this unmeasured confounder rather than any pathway that reducing anxiety could exploit. Fourth, students are nested in classes and teachers, but no class or teacher identifiers were recorded; class- and teacher-level variance, to which the teacher-appreciation items refer by definition, is therefore conflated with student-level conditional dependencies, and we could not compute intraclass correlations. Fifth, the polychoric correlations assume latent bivariate normality of the ordinal items, an assumption our descriptive checks do not test; violations would propagate into the network and the simulated effects. Sixth, the simulation used the regularized precision matrix, so graphical LASSO shrinkage propagates into the simulated effects, and the bootstrap intervals for the alleviation effects were computed under rejection sampling of non-positive-definite resampled correlation matrices. Finally, the sample came from a single mid-ranked Chinese university and was concentrated in first-year students, and all measures were self-reports; population-level generalization and the role of gender or year of study therefore remain open questions. Field experiments that prepare students for speaking tasks and track enjoyment before and after would be the natural next step, alongside replications that translate the identified nodes into real instructional designs.

## 5 Conclusion

Anxiety and enjoyment in the foreign language classroom are linked but far from mirror images, and their coupling in the present study can be located at the level of individual classroom experiences. In a network of 3,957 Chinese non-English-major college students, social and teacher-related enjoyment items were most central, and among anxiety items, panic about unprepared speaking carried the highest influence. Simulated node conditioning went beyond centrality: across two complementary criteria, distributional reconfiguration and signed predicted gain, the anxiety experiences most tightly coupled to enjoyment were speaking-related ones, panic without preparation and nervousness when speaking, whereas the leading bridge node was practically inert. The study therefore complements the structural criterion of “most connected” with the model-implied criterion of “most coupled in simulation,” which speaks directly to the needs of teachers who must decide where to spend their limited classroom time. The practical message is compact and stated as a hypothesis: preparing students for speaking tasks (through preparation time, scaffolding, and low-stakes speaking practice) is the classroom lever our simulations most consistently associate with enjoyment, and it is inexpensive to test. These findings offer a concrete starting point for classroom-focused experimental work and a methodological template in which hypotheses about intervention targets are generated from simulated consequence rather than from structural connectedness alone.

## Data Availability

The raw data supporting the conclusions of this article will be made available by the authors, without undue reservation.
