# Supplementary material for: Which classroom anxiety experience should teachers target first? A network-simulation study of foreign language classroom anxiety and enjoyment
Source: Front Psychiatry. 2026 Oct 13;17:1994670. doi: 10.3389/fpsyt.2026.1994670 (PMC13711261; doi:10.3389/fpsyt.2026.1994670)
Supplement: Supplementary file 1 [file Table1.docx]

**Supplementary Analyses for the Revised Manuscript**

**Contents**

[Section SA1. Validation evidence for the GGMNIRA framework	3](#_Toc241046446)

[Section SA2. Principal-direction decomposition of the simulated shifts (Table RA1)	5](#_Toc241046447)

[Table RA1. Principal-direction decomposition of each anxiety node's propagated shift of the nine enjoyment variables (*δ* = 1).	6](#_Toc241046448)

[Section SA3. Reverse-coding documentation and zero-order evidence for ANX5 (Table RA2)	7](#_Toc241046449)

[Table RA2. Item psychometrics of the eight S-FLCAS anxiety items and zero-order polychoric correlations with ANX5.	8](#_Toc241046450)

[Section SA4. Bootstrap inference for the alleviation effects (Table RA3; Figure RA1)	9](#_Toc241046451)

[Table RA3. Nonparametric bootstrap inference for the alleviation effects (500 accepted resamples; polychoric/EBICglasso pipeline).	10](#_Toc241046452)

[Section SA5. Full polychoric correlation matrix (Table RA4)	12](#_Toc241046453)

[Table RA4. Full 17 × 17 polychoric correlation matrix used by the network model.	13](#_Toc241046454)

[Section SA6. Sensitivity analysis excluding ANX5 (Table RA5)	14](#_Toc241046455)

[Table RA5. Sensitivity analysis excluding ANX5: KL and alleviation effects, 16-node versus 17-node network.	14](#_Toc241046456)

[Section SA7. Topological overlap and composite-node re-analysis (Tables RA6–RA7)	15](#_Toc241046457)

[Table RA6a. Goldbricker pair-wise comparisons; no pair is flagged at the default decision rule.	16](#_Toc241046458)

[Table RA6b. Unique variable analysis (UVA): locally dependent clusters flagged.	16](#_Toc241046459)

[Table RA7. Composite-node re-analysis (ENJ1–ENJ3 combined into ENJ_T; 15-node network) versus the main 17-node analysis.	17](#_Toc241046460)

[Section SA8. Community structure, network density, and centrality difference tests (Tables RA8–RA9)	18](#_Toc241046461)

[Table RA8. Community assignments from exploratory graph analysis (EGA) and walktrap clustering versus the two-construct partition (walktrap modularity Q = 0.494).	19](#_Toc241046462)

[Table RA9. Bootstrap centrality difference-test summary (5,000 resamples) and ANX5 bridge-edge composition.	19](#_Toc241046463)

[Section SA9. Benchmark regression analysis (Table RA10)	20](#_Toc241046464)

[Table RA10. Benchmark multiple regression of the enjoyment composite (unit-weighted sum) on the eight anxiety items; all variables standardized (*R²* = 0.078).	20](#_Toc241046465)

[Section SA10. Reverse-direction simulation (Table RA11)	21](#_Toc241046466)

[Table RA11. Reverse-direction simulation: conditioning each enjoyment node (*δ* = ±1) and predicting the anxiety construct.	21](#_Toc241046467)

[Section SA11. Simulated effects on the original 1–5 scale; extrapolation (Table RA12)	22](#_Toc241046468)

[Table RA12. Simulated alleviation effects (*δ* = −1) re-expressed on the original 1–5 response scale: per-item raw-point shifts, raw total shift, and share of the observed total-score *SD* (8.30).	23](#_Toc241046469)

[Section SA12. Minimum effective manipulation strength (Table RA13)	24](#_Toc241046470)

[Table RA13. Minimum effective manipulation strength, a monotone transformation of the KL value (the column removed from main-text Table 2).	24](#_Toc241046471)

# Section SA1. Validation evidence for the GGMNIRA framework

The GGMNIRA framework, its stability procedures, and the interpretive benchmarks used in the main text derive from Wu, Wang, and Liu (2026, arXiv:2607.00915). We summarize here the validation evidence contained in that source, so that the benchmarks do not rest on the preprint's own empirical output alone. (1) The correlation-stability (CS) coefficient and its case-dropping implementation are inherited from Epskamp, Borsboom, and Fried (2018), the standard accuracy framework in network psychometrics; the preprint extends the same machinery to KL rankings. (2) The 0.25/0.40 interpretive thresholds for the KL-CS coefficient were calibrated against a known-truth null: in the preprint's simulation of 8,000 datasets generated from known structures, the 3,051-dataset subset in which all true KL values were analytically equal (the equal condition) provides the null distribution, and approximately 75% of CS coefficients in that subset fell below 0.25 and 95% below 0.40; the thresholds are thus the 75th and 95th percentiles of an independent simulation null distribution, not a self-evaluation of the method's output. (3) The KL pairwise difference test was calibrated in the same work: empirical type-I error rates were 0.047–0.049 at *n* ≥ 500 (nominal 0.05), with power increasing in *n* and structural heterogeneity (up to 0.817 at *n* = 5,000 under full random rewiring). (4) An 81,000-network appendix relates KL rankings to strength centrality (correlations 0.69–0.97 depending on between-dimension connectivity), supporting the interpretation of GGMNIRA as a higher-order generalization of centrality. We add one caveat of our own: these simulations calibrate the estimation pipeline against model-internal analytic truth; recovery of an external, dynamic ground truth remains an open problem for the entire in-silico intervention literature (cf. Dablander & Hinne, 2019), and we therefore present our rankings as model-implied hypotheses.

# Section SA2. Principal-direction decomposition of the simulated shifts (Table RA1)

Each anxiety node's propagated shift of the nine enjoyment variables (the corresponding column of the propagation matrix *M*, restricted to the enjoyment block) was decomposed into (i) its signed projection onto the first principal direction of the enjoyment marginal covariance matrix and (ii) the orthogonal, profile component. The first principal direction accounts for 85.1% of the enjoyment covariance (loadings 0.319–0.343, a general enjoyment factor). Table RA1 reports, per node: the KL divergence (*δ* = 1), the signed PC1 projection, the share of the squared shift lying on PC1, the norm of the orthogonal component, and the signed sum of the shift (the alleviation effect). Across nodes, KL correlates *r* = 0.92 with the orthogonal component and *r* = 0.84 with the absolute PC1 projection, confirming that the KL criterion is driven by the profile-reshaping component and prices shifts along the main enjoyment direction cheapest (the PC1 direction costs 1/604 of the most expensive direction per unit squared shift).

Table RA1. Principal-direction decomposition of each anxiety node's propagated shift of the nine enjoyment variables (*δ* = 1).

| **Node** | **Label** | **KL (*δ* = 1)** | **Signed PC1 projection** | **Share of squared shift on PC1** | **Profile-component norm** | **Signed sum (alleviation effect)** |
| --- | --- | --- | --- | --- | --- | --- |
| ANX1 | Fear of mistakes | 0.580 | -1.303 | 0.778 | 0.696 | 3.922 |
| ANX2 | Panic without preparation | 1.683 | -2.412 | 0.806 | 1.184 | 7.260 |
| ANX3 | Reluctant to volunteer | 1.229 | -1.834 | 0.740 | 1.086 | 5.522 |
| ANX4 | Anxious despite preparation | 0.633 | -2.215 | 0.952 | 0.497 | 6.652 |
| ANX5 | Low speaking confidence | 0.033 | 0.644 | 0.992 | 0.057 | -1.933 |
| ANX6 | Heart pounds when called | 0.953 | -2.175 | 0.882 | 0.797 | 6.539 |
| ANX7 | Others better at English | 0.686 | -1.763 | 0.862 | 0.706 | 5.303 |
| ANX8 | Nervous when speaking | 1.555 | -3.121 | 0.920 | 0.918 | 9.379 |

# Section SA3. Reverse-coding documentation and zero-order evidence for ANX5 (Table RA2)

Exactly one anxiety item is reverse-worded: ANX5 ("I feel confident speaking English in class"). It was recoded before analysis so that higher scores indicate greater anxiety, using the syntax:

Evidence that the coding is correct: if ANX5 had been left unreversed, or reversed twice, its zero-order correlations with the other seven anxiety items would be negative. In the analyzed data these correlations are uniformly positive (polychoric *r* = 0.317–0.421; Pearson *r* = 0.278–0.372; Spearman *r* = 0.285–0.384). The positive association between ANX5 and the enjoyment items (e.g., ANX5–ENJ6: polychoric *r* = 0.190, Pearson *r* = 0.173) is likewise a zero-order feature of the data, not a product of network estimation. ANX5 is nevertheless psychometrically the weakest anxiety item: its corrected item-total correlation is 0.367 (versus 0.784–0.859 for the other seven items), and the scale *α* rises from 0.934 to 0.952 when ANX5 is removed. ANX5 response distribution: 1 = 841, 2 = 891, 3 = 1,519, 4 = 565, 5 = 141.

Table RA2. Item psychometrics of the eight S-FLCAS anxiety items and zero-order polychoric correlations with ANX5.

| **Item** | **Label** | **Corrected item-total *r*** | ***α* if item deleted** | **Mean** | ***SD*** | **Polychoric *r* with ANX5** |
| --- | --- | --- | --- | --- | --- | --- |
| ANX1 | Fear of mistakes | 0.784 | 0.924 | 2.542 | 1.184 | 0.317 |
| ANX2 | Panic without preparation | 0.859 | 0.919 | 2.518 | 1.189 | 0.353 |
| ANX3 | Reluctant to volunteer | 0.818 | 0.922 | 2.617 | 1.202 | 0.321 |
| ANX4 | Anxious despite preparation | 0.836 | 0.921 | 2.332 | 1.061 | 0.421 |
| ANX5 | Low speaking confidence | 0.367 | 0.952 | 2.564 | 1.082 | – |
| ANX6 | Heart pounds when called | 0.847 | 0.920 | 2.437 | 1.116 | 0.414 |
| ANX7 | Others better at English | 0.816 | 0.922 | 2.463 | 1.100 | 0.386 |
| ANX8 | Nervous when speaking | 0.855 | 0.919 | 2.375 | 1.070 | 0.362 |

# Section SA4. Bootstrap inference for the alleviation effects (Table RA3; Figure RA1)

Nonparametric bootstrap inference for the alleviation effects used 500 accepted resamples of the data (985 proposals; proposals whose polychoric correlation matrix was not positive definite were rejected and redrawn, consistent with the rejection-sampling strategy disclosed for the original KL bootstrap). Each resample network was re-estimated with the identical pipeline as the main analysis (EBICglasso, *γ* = 0.5, cor_auto polychoric correlations), and the alleviation effect was recomputed from the resampled propagation matrix. All eight bootstrap 95% confidence intervals (percentile method, Hyndman–Fan type 6) exclude zero, and each contains the corresponding full-sample point estimate. Pairwise difference tests (bootstrap 95% CI of the pairwise difference excluding zero) show 24 of the 28 unique comparisons significant; under this signed criterion, ANX8 exceeds ANX2 (95% CI of difference [0.76, 3.59]) and ANX3 ([2.51, 5.44]) significantly, and ANX2 exceeds ANX3 ([0.58, 3.18]). The four non-significant pairs are ANX2–ANX4, ANX2–ANX6, ANX4–ANX6, and ANX3–ANX7. No multiplicity correction was applied (see Section 2.3 of the main text). The same bootstrap reproduces the published KL difference-test pattern (22 of 28 pairs significant, with the three near-boundary pairs differing from the original Pearson-metric run disclosed in the original Supplementary Materials).

Table RA3. Nonparametric bootstrap inference for the alleviation effects (500 accepted resamples; polychoric/EBICglasso pipeline).

| **Node** | **Label** | **Point estimate** | **Bootstrap mean** | **Bootstrap *SD*** | **95% CI lower** | **95% CI upper** |
| --- | --- | --- | --- | --- | --- | --- |
| ANX1 | Fear of mistakes | 3.922 | 3.929 | 0.665 | 2.614 | 5.215 |
| ANX2 | Panic without preparation | 7.260 | 7.297 | 1.066 | 5.245 | 9.493 |
| ANX3 | Reluctant to volunteer | 5.522 | 5.559 | 0.765 | 4.024 | 7.088 |
| ANX4 | Anxious despite preparation | 6.652 | 6.670 | 0.752 | 5.136 | 8.206 |
| ANX5 | Low speaking confidence | -1.933 | -1.947 | 0.277 | -2.470 | -1.368 |
| ANX6 | Heart pounds when called | 6.539 | 6.656 | 0.857 | 5.055 | 8.589 |
| ANX7 | Others better at English | 5.303 | 5.260 | 0.717 | 3.836 | 6.764 |
| ANX8 | Nervous when speaking | 9.379 | 9.489 | 1.055 | 7.441 | 11.641 |

**Figure RA1. Model-implied change in the sum of the nine enjoyment latent variables per 1-*SD* simulated alleviation of each anxiety node, with 95% nonparametric bootstrap confidence intervals (identical to revised main-text Figure 3).**

# Section SA5. Full polychoric correlation matrix (Table RA4)

The complete 17 × 17 polychoric correlation matrix used by the network model is provided for independent verification of the recoding and of every edge discussed in the text.

Table RA4. Full 17 × 17 polychoric correlation matrix used by the network model.

|  | **ANX1** | **ANX2** | **ANX3** | **ANX4** | **ANX5** | **ANX6** | **ANX7** | **ANX8** | **ENJ1** | **ENJ2** | **ENJ3** | **ENJ4** | **ENJ5** | **ENJ6** | **ENJ7** | **ENJ8** | **ENJ9** |
| --- | --- | --- | --- | --- | --- | --- | --- | --- | --- | --- | --- | --- | --- | --- | --- | --- | --- |
| ANX1 | 1.000 | 0.820 | 0.763 | 0.746 | 0.317 | 0.749 | 0.717 | 0.770 | -0.031 | -0.028 | -0.032 | -0.162 | -0.124 | -0.241 | -0.160 | -0.173 | -0.171 |
| ANX2 | 0.820 | 1.000 | 0.827 | 0.799 | 0.353 | 0.822 | 0.789 | 0.829 | -0.046 | -0.043 | -0.047 | -0.192 | -0.150 | -0.270 | -0.212 | -0.213 | -0.208 |
| ANX3 | 0.763 | 0.827 | 1.000 | 0.775 | 0.321 | 0.785 | 0.759 | 0.782 | -0.029 | -0.021 | -0.024 | -0.197 | -0.148 | -0.281 | -0.234 | -0.235 | -0.228 |
| ANX4 | 0.746 | 0.799 | 0.775 | 1.000 | 0.421 | 0.794 | 0.767 | 0.812 | -0.135 | -0.143 | -0.142 | -0.213 | -0.203 | -0.235 | -0.195 | -0.200 | -0.207 |
| ANX5 | 0.317 | 0.353 | 0.321 | 0.421 | 1.000 | 0.414 | 0.386 | 0.362 | 0.139 | 0.135 | 0.131 | 0.158 | 0.147 | 0.190 | 0.146 | 0.155 | 0.152 |
| ANX6 | 0.749 | 0.822 | 0.785 | 0.794 | 0.414 | 1.000 | 0.782 | 0.843 | -0.089 | -0.089 | -0.094 | -0.210 | -0.174 | -0.253 | -0.202 | -0.209 | -0.210 |
| ANX7 | 0.717 | 0.789 | 0.759 | 0.767 | 0.386 | 0.782 | 1.000 | 0.820 | -0.068 | -0.068 | -0.069 | -0.196 | -0.159 | -0.266 | -0.194 | -0.196 | -0.198 |
| ANX8 | 0.770 | 0.829 | 0.782 | 0.812 | 0.362 | 0.843 | 0.820 | 1.000 | -0.122 | -0.131 | -0.133 | -0.256 | -0.213 | -0.293 | -0.226 | -0.238 | -0.240 |
| ENJ1 | -0.031 | -0.046 | -0.029 | -0.135 | 0.139 | -0.089 | -0.068 | -0.122 | 1.000 | 0.974 | 0.975 | 0.868 | 0.855 | 0.695 | 0.738 | 0.753 | 0.789 |
| ENJ2 | -0.028 | -0.043 | -0.021 | -0.143 | 0.135 | -0.089 | -0.068 | -0.131 | 0.974 | 1.000 | 0.981 | 0.863 | 0.850 | 0.687 | 0.726 | 0.740 | 0.777 |
| ENJ3 | -0.032 | -0.047 | -0.024 | -0.142 | 0.131 | -0.094 | -0.069 | -0.133 | 0.975 | 0.981 | 1.000 | 0.870 | 0.858 | 0.694 | 0.740 | 0.752 | 0.787 |
| ENJ4 | -0.162 | -0.192 | -0.197 | -0.213 | 0.158 | -0.210 | -0.196 | -0.256 | 0.868 | 0.863 | 0.870 | 1.000 | 0.894 | 0.818 | 0.803 | 0.827 | 0.841 |
| ENJ5 | -0.124 | -0.150 | -0.148 | -0.203 | 0.147 | -0.174 | -0.159 | -0.213 | 0.855 | 0.850 | 0.858 | 0.894 | 1.000 | 0.835 | 0.839 | 0.862 | 0.881 |
| ENJ6 | -0.241 | -0.270 | -0.281 | -0.235 | 0.190 | -0.253 | -0.266 | -0.293 | 0.695 | 0.687 | 0.694 | 0.818 | 0.835 | 1.000 | 0.854 | 0.862 | 0.861 |
| ENJ7 | -0.160 | -0.212 | -0.234 | -0.195 | 0.146 | -0.202 | -0.194 | -0.226 | 0.738 | 0.726 | 0.740 | 0.803 | 0.839 | 0.854 | 1.000 | 0.903 | 0.913 |
| ENJ8 | -0.173 | -0.213 | -0.235 | -0.200 | 0.155 | -0.209 | -0.196 | -0.238 | 0.753 | 0.740 | 0.752 | 0.827 | 0.862 | 0.862 | 0.903 | 1.000 | 0.941 |
| ENJ9 | -0.171 | -0.208 | -0.228 | -0.207 | 0.152 | -0.210 | -0.198 | -0.240 | 0.789 | 0.777 | 0.787 | 0.841 | 0.881 | 0.861 | 0.913 | 0.941 | 1.000 |

# Section SA6. Sensitivity analysis excluding ANX5 (Table RA5)

The network and both simulation criteria were re-estimated on the 16-node dataset excluding ANX5 (same pipeline as the main analysis). The KL ranking of the remaining seven anxiety nodes is identical to the main analysis (Spearman *ρ* = 1.00), and the alleviation ranking is nearly identical (*ρ* = 0.96; only ANX4 and ANX6 exchange ranks 3–4). Nervous when speaking (ANX8) remains the largest signed alleviation effect (+9.37), and the top KL tier (ANX2, ANX8, ANX3) is unchanged. No substantive conclusion depends on ANX5.

Table RA5. Sensitivity analysis excluding ANX5: KL and alleviation effects, 16-node versus 17-node network.

| **Node** | **Label** | **KL (17-node)** | **KL (16-node)** | **Alleviation (17)** | **Alleviation (16)** | **Rank KL 17** | **Rank KL 16** | **Rank AL 17** | **Rank AL 16** |
| --- | --- | --- | --- | --- | --- | --- | --- | --- | --- |
| ANX2 | Panic without preparation | 1.683 | 1.682 | 7.260 | 7.254 | 1 | 1 | 2 | 2 |
| ANX8 | Nervous when speaking | 1.555 | 1.572 | 9.379 | 9.368 | 2 | 2 | 1 | 1 |
| ANX3 | Reluctant to volunteer | 1.229 | 1.224 | 5.522 | 5.515 | 3 | 3 | 5 | 5 |
| ANX6 | Heart pounds when called | 0.953 | 0.986 | 6.539 | 6.614 | 4 | 4 | 4 | 3 |
| ANX7 | Others better at English | 0.686 | 0.681 | 5.303 | 5.262 | 5 | 5 | 6 | 6 |
| ANX4 | Anxious despite preparation | 0.633 | 0.609 | 6.652 | 6.522 | 6 | 6 | 3 | 4 |
| ANX1 | Fear of mistakes | 0.580 | 0.579 | 3.922 | 3.921 | 7 | 7 | 7 | 7 |

# Section SA7. Topological overlap and composite-node re-analysis (Tables RA6–RA7)

The three teacher-appreciation items (ENJ1–ENJ3) intercorrelate at polychoric *r* = 0.974–0.981 (Pearson 0.952–0.964). Two standard procedures were applied. Unique variable analysis (UVA; Christensen et al., 2023) flags the ENJ1–ENJ3 triplet as locally dependent (and additionally the smaller clusters ENJ7–ENJ8–ENJ9, ENJ4–ENJ5, and ANX1–ANX2). The goldbricker procedure flags no pairs at its default decision rule; we report this divergence transparently and note that with *N* = 3,957 even very small model differences become statistically significant, which affects the two procedures differently. Following the reviewer's remedy, ENJ1–ENJ3 were combined into a single teacher-appreciation composite node (ENJ_T, the item mean), and the entire pipeline was re-run on the resulting 15-node dataset. Both rankings are preserved (KL and alleviation: Spearman *ρ* = 0.98 versus the main analysis); the top KL tier (ANX2, ANX8, ANX3) is unchanged; ANX8 remains the largest signed alleviation effect, attenuated from +9.38 to +7.70 (−17.9%), a direct quantification of how much the redundant triplet contributes to the headline magnitude. All substantive conclusions are unchanged.

Table RA6a. Goldbricker pair-wise comparisons; no pair is flagged at the default decision rule.

| **Node 1** | **Node 2** | **Proportion of significant differences** | **Polychoric *r*** |
| --- | --- | --- | --- |
| ENJ2 | ENJ1 | 0.800 | 0.974 |
| ENJ3 | ENJ1 | 0.467 | 0.975 |
| ENJ3 | ENJ2 | 0.800 | 0.981 |
| ENJ8 | ENJ7 | 0.533 | 0.922 |
| ENJ9 | ENJ7 | 0.533 | 0.927 |
| ENJ9 | ENJ8 | 0.400 | 0.960 |
| ENJ5 | ENJ4 | 0.867 | 0.926 |
| ANX2 | ANX1 | 0.800 | 0.820 |
| ANX8 | ANX6 | 0.867 | 0.843 |

Polychoric *r* values are goldbricker's internal pairwise estimates, except for the three ENJ1–ENJ3 pairs, which are taken from the full 17 × 17 polychoric correlation matrix (Table RA4) so that a single estimator is used consistently throughout this document; goldbricker's internal estimates for these pairs were 0.995, 0.996, and 0.997.

Table RA6b. Unique variable analysis (UVA): locally dependent clusters flagged.

| **Cluster** | **Items** | **Content** |
| --- | --- | --- |
| 1 | ENJ1, ENJ2, ENJ3 | Teacher-appreciation triplet |
| 2 | ENJ7, ENJ8, ENJ9 | Social-enjoyment triplet |
| 3 | ENJ4, ENJ5 | Personal-enjoyment pair |
| 4 | ANX1, ANX2 | Anxiety pair |

Table RA7. Composite-node re-analysis (ENJ1–ENJ3 combined into ENJ_T; 15-node network) versus the main 17-node analysis.

| **Node** | **Label** | **KL (17)** | **KL (15)** | **Alleviation (17)** | **Alleviation (15)** | **Alleviation Δ%** | **Rank KL 17** | **Rank KL 15** | **Rank AL 17** | **Rank AL 15** |
| --- | --- | --- | --- | --- | --- | --- | --- | --- | --- | --- |
| ANX2 | Panic without preparation | 1.683 | 2.807 | 7.260 | 6.226 | -14.240 | 1 | 1 | 2 | 2 |
| ANX8 | Nervous when speaking | 1.555 | 2.570 | 9.379 | 7.697 | -17.940 | 2 | 2 | 1 | 1 |
| ANX3 | Reluctant to volunteer | 1.229 | 1.920 | 5.522 | 4.919 | -10.927 | 3 | 3 | 5 | 5 |
| ANX6 | Heart pounds when called | 0.953 | 1.676 | 6.539 | 5.460 | -16.502 | 4 | 4 | 4 | 3 |
| ANX7 | Others better at English | 0.686 | 1.173 | 5.303 | 4.392 | -17.186 | 5 | 6 | 6 | 6 |
| ANX4 | Anxious despite preparation | 0.633 | 1.177 | 6.652 | 5.122 | -22.992 | 6 | 5 | 3 | 4 |
| ANX1 | Fear of mistakes | 0.580 | 0.980 | 3.922 | 3.386 | -13.655 | 7 | 7 | 7 | 7 |
| ANX5 | Low speaking confidence | 0.033 | 0.052 | -1.933 | -1.602 | 17.135 | 8 | 8 | 8 | 8 |

# Section SA8. Community structure, network density, and centrality difference tests (Tables RA8–RA9)

Data-driven community detection was used to verify the two-construct partition assumed by the bridge indices. Exploratory graph analysis (EGA) and walktrap clustering (modularity Q = 0.494) both return three communities: {ANX1–ANX8}, {ENJ1–ENJ3}, and {ENJ4–ENJ9}, with no node misassigned across the anxiety/enjoyment constructs, supporting the bridge specification. The network is moderately dense: 83 of 136 possible edges are non-zero (49 within-construct, 34 cross-construct). Bootstrap centrality difference tests (5,000 resamples, original supplement) show that ENJ9's expected influence exceeds that of 12 of the other 16 nodes and ENJ3's exceeds 9 of 16; the case-dropping coefficients of 0.75 equal the maximum proportion tested and should be read as "at least 0.75." The outlying bridge EI of ANX5 (*z* = 3.30) is driven essentially by a single edge: ANX5–ENJ6 (*w* = 0.159) contributes 61.6% of ANX5's total absolute bridge weight.

Table RA8. Community assignments from exploratory graph analysis (EGA) and walktrap clustering versus the two-construct partition (walktrap modularity Q = 0.494).

| **Node** | **Label** | **Construct** | **EGA community** | **Walktrap community** |
| --- | --- | --- | --- | --- |
| ANX1 | Fear of mistakes | Anxiety | 1 | 2 |
| ANX2 | Panic without preparation | Anxiety | 1 | 2 |
| ANX3 | Reluctant to volunteer | Anxiety | 1 | 2 |
| ANX4 | Anxious despite preparation | Anxiety | 1 | 2 |
| ANX5 | Low speaking confidence | Anxiety | 1 | 2 |
| ANX6 | Heart pounds when called | Anxiety | 1 | 2 |
| ANX7 | Others better at English | Anxiety | 1 | 2 |
| ANX8 | Nervous when speaking | Anxiety | 1 | 2 |
| ENJ1 | Teacher encourages us | Enjoyment | 2 | 3 |
| ENJ2 | Teacher is kind | Enjoyment | 2 | 3 |
| ENJ3 | Teacher is supportive | Enjoyment | 2 | 3 |
| ENJ4 | Enjoy learning English | Enjoyment | 3 | 1 |
| ENJ5 | Learn interesting things | Enjoyment | 3 | 1 |
| ENJ6 | Proud of my English | Enjoyment | 3 | 1 |
| ENJ7 | Close study group | Enjoyment | 3 | 1 |
| ENJ8 | Laughter in class | Enjoyment | 3 | 1 |
| ENJ9 | Shared jokes and topics | Enjoyment | 3 | 1 |

Table RA9. Bootstrap centrality difference-test summary (5,000 resamples) and ANX5 bridge-edge composition.

| **Quantity** | **Value** | **Detail** |
| --- | --- | --- |
| ENJ9 expected influence | EI = 1.195 | Significantly greater than 12 of the other 16 nodes (ANX1, ANX3–ANX8, ENJ2, ENJ4–ENJ7) |
| ENJ3 expected influence | EI = 1.132 | Significantly greater than 9 of the other 16 nodes (ANX1, ANX3, ANX5, ANX7, ENJ2, ENJ4–ENJ7) |
| ANX5 bridge expected influence | BEI = 0.189, *z* = 3.298 | Significantly greater than all 16 other nodes |
| ANX5–ENJ6 edge | *w* = 0.159 | 61.6% of ANX5's total absolute bridge weight (\|*w*\| total = 0.258) |
| Network density | 83 of 136 edges non-zero | 49 within-construct (ANX 26/28; ENJ 23/36), 34 cross-construct (of 72) |

# Section SA9. Benchmark regression analysis (Table RA10)

As a transparency benchmark, the enjoyment composite (unit-weighted sum) was regressed on the eight anxiety items (all variables standardized). The model explains *R²* = 0.078 of the variance; the largest standardized coefficient belongs to ANX5 (*β* = +0.258), the reverse-worded item whose positive association with enjoyment we attribute to a response-style artifact, while ANX2, ANX3, and ANX7 do not reach significance. The ranking of the absolute regression coefficients is uncorrelated with the alleviation ranking (Spearman *ρ* = −0.024). A latent enjoyment factor score as outcome yields the same pattern (*R²* = 0.076). The comparison clarifies what the simulation adds beyond regression: item-level profiles of predicted change across all nine enjoyment variables, a distribution-level (KL) summary, and propagation through indirect paths that a composite regression absorbs into its coefficients and error term.

Table RA10. Benchmark multiple regression of the enjoyment composite (unit-weighted sum) on the eight anxiety items; all variables standardized (*R²* = 0.078).

| **Predictor** | **Label** | ***β*** | ***SE*** | **95% CI lower** | **95% CI upper** | ***p*** |
| --- | --- | --- | --- | --- | --- | --- |
| ANX5 | Low speaking confidence | 0.258 | 0.017 | 0.226 | 0.291 | < 0.001 |
| ANX8 | Nervous when speaking | -0.132 | 0.032 | -0.194 | -0.071 | < 0.001 |
| ANX4 | Anxious despite preparation | -0.117 | 0.028 | -0.172 | -0.062 | < 0.001 |
| ANX1 | Fear of mistakes | 0.070 | 0.026 | 0.020 | 0.121 | 0.007 |
| ANX6 | Heart pounds when called | -0.055 | 0.030 | -0.113 | 0.003 | 0.063 |
| ANX2 | Panic without preparation | 0.016 | 0.032 | -0.046 | 0.078 | 0.623 |
| ANX7 | Others better at English | -0.012 | 0.027 | -0.066 | 0.041 | 0.649 |
| ANX3 | Reluctant to volunteer | -0.008 | 0.028 | -0.063 | 0.047 | 0.773 |

# Section SA10. Reverse-direction simulation (Table RA11)

For comparison, each enjoyment node was conditioned in turn (*δ* = ±1) and the predicted change in the sum of the eight standardized anxiety variables and the KL divergence of the anxiety marginal distribution were recorded. The reverse direction is not a mirror image of the forward direction: under the linear framework the reverse and forward propagation coefficients satisfy *m_rev = m_fwd · (θ_jj/θ_ii)*, and because the teacher-appreciation items have very large precision diagonal elements (*θ* = 46–53), the reverse KL values are dominated by ENJ1–ENJ3 (KL = 125.6, 105.3, 95.6). This asymmetry is a mathematical property of the estimated linear system and carries no causal interpretation on cross-sectional data.

Table RA11. Reverse-direction simulation: conditioning each enjoyment node (*δ* = ±1) and predicting the anxiety construct.

| **Node** | **Label** | **Reverse alleviation (Δ anxiety sum)** | **Reverse KL** | **KL rank** |
| --- | --- | --- | --- | --- |
| ENJ3 | Teacher is supportive | 22.080 | 125.615 | 1 |
| ENJ2 | Teacher is kind | 19.503 | 105.260 | 2 |
| ENJ1 | Teacher encourages us | 19.276 | 95.612 | 3 |
| ENJ9 | Shared jokes and topics | 19.326 | 15.195 | 4 |
| ENJ8 | Laughter in class | 15.645 | 9.848 | 5 |
| ENJ5 | Learn interesting things | 9.750 | 5.282 | 6 |
| ENJ4 | Enjoy learning English | 10.781 | 5.165 | 7 |
| ENJ7 | Close study group | 9.859 | 3.993 | 8 |
| ENJ6 | Proud of my English | 8.797 | 2.841 | 9 |

# Section SA11. Simulated effects on the original 1–5 scale; extrapolation (Table RA12)

To let readers judge plausibility, the simulated alleviation effects are re-expressed on the original response scale by multiplying each enjoyment item's standardized shift by its observed *SD*. Alleviating ANX8 by one marginal *SD* corresponds to a predicted gain of ≈ +9.7 raw points on the 9–45 enjoyment total (about 1.17 observed total-score *SD*s; observed total *SD* = 8.30). We stress that these are linear model extrapolations: the manipulation strengths at which effects become "effective" under the framework's geometric criterion (e.g., *δ* = 2.24 for ANX2, corresponding to ≈ 2.7 raw points on a 1–5 item with mean 2.52) fall below the floor of the response scale, and a node's own marginal mean shift (*δ·θ_jj*) amplifies the extrapolation further. The rankings, not the absolute magnitudes, carry the interpretation.

Table RA12. Simulated alleviation effects (*δ* = −1) re-expressed on the original 1–5 response scale: per-item raw-point shifts, raw total shift, and share of the observed total-score *SD* (8.30).

| **Node alleviated** | **ENJ1** | **ENJ2** | **ENJ3** | **ENJ4** | **ENJ5** | **ENJ6** | **ENJ7** | **ENJ8** | **ENJ9** | **Total (9–45 scale)** | **% of observed total *SD*** |
| --- | --- | --- | --- | --- | --- | --- | --- | --- | --- | --- | --- |
| ANX1 | 0.133 | 0.125 | 0.132 | 0.558 | 0.456 | 0.843 | 0.611 | 0.630 | 0.605 | 4.092 | 49.315% |
| ANX2 | 0.287 | 0.275 | 0.286 | 1.007 | 0.842 | 1.476 | 1.137 | 1.149 | 1.107 | 7.565 | 91.158% |
| ANX3 | 0.140 | 0.121 | 0.133 | 0.766 | 0.636 | 1.171 | 0.954 | 0.949 | 0.905 | 5.774 | 69.581% |
| ANX4 | 0.511 | 0.517 | 0.514 | 0.906 | 0.765 | 1.044 | 0.861 | 0.877 | 0.874 | 6.869 | 82.770% |
| ANX5 | -0.197 | -0.194 | -0.195 | -0.214 | -0.222 | -0.273 | -0.233 | -0.232 | -0.226 | -1.987 | -23.942% |
| ANX6 | 0.370 | 0.364 | 0.370 | 0.910 | 0.760 | 1.174 | 0.944 | 0.957 | 0.935 | 6.785 | 81.757% |
| ANX7 | 0.277 | 0.272 | 0.278 | 0.725 | 0.615 | 1.000 | 0.784 | 0.789 | 0.768 | 5.509 | 66.386% |
| ANX8 | 0.628 | 0.622 | 0.630 | 1.290 | 1.094 | 1.577 | 1.281 | 1.304 | 1.281 | 9.708 | 116.988% |

# Section SA12. Minimum effective manipulation strength (Table RA13)

The minimum effective manipulation strength column removed from main-text Table 2 is reported here for completeness. Because KL scales with *δ²* for a mean shift, the minimum effective *δ* is a monotone transformation of the KL value, *δ_min = √(χ²(0.95, df = 9) / (2·KL)) = √(8.46/KL)*, and carries no information beyond Table 2. The underlying effectiveness criterion is a geometric convention (whether the shifted mean vector falls outside the 95% probability ellipsoid of the baseline distribution), not a hypothesis test or an effect-size benchmark; it is independent of sample size. All values in this column exceed the observed range of the items (see Section SA11).

Table RA13. Minimum effective manipulation strength, a monotone transformation of the KL value (the column removed from main-text Table 2).

| **Node** | **Label** | **KL (*δ* = 1, nats)** | **Minimum effective *δ* = *√(8.46/KL)*** |
| --- | --- | --- | --- |
| ANX1 | Fear of mistakes | 0.580 | 3.820 |
| ANX2 | Panic without preparation | 1.683 | 2.242 |
| ANX3 | Reluctant to volunteer | 1.229 | 2.623 |
| ANX4 | Anxious despite preparation | 0.633 | 3.656 |
| ANX5 | Low speaking confidence | 0.033 | 15.96 |
| ANX6 | Heart pounds when called | 0.953 | 2.979 |
| ANX7 | Others better at English | 0.686 | 3.512 |
| ANX8 | Nervous when speaking | 1.555 | 2.333 |
