# Supplementary material for: Which classroom anxiety experience should teachers target first? A network-simulation study of foreign language classroom anxiety and enjoyment
Source: Front Psychiatry. 2026 Oct 13;17:1994670. doi: 10.3389/fpsyt.2026.1994670 (PMC13711261; doi:10.3389/fpsyt.2026.1994670)
Supplement: Supplementary file 2 [file Table2.docx]

**S1. Centrality Stability: Case-Dropping Subset Bootstrap**

Stability of the centrality indices was assessed with the case-dropping subset bootstrap (5,000 resamples). The correlation stability (CS) coefficient quantifies the maximum proportion of cases that can be dropped while the centrality order in the subset correlates at *r* ≥ 0.70 with the original order with 95% probability. Values above 0.25 are acceptable and values above 0.50 are good (15).

Figure S1. Case-dropping stability curves for expected influence (EI) and bridge expected influence (BEI), with 95% quantile bands.

**S2. Accuracy of Edge Weights: 95% Bootstrap Confidence Intervals**

Edge-weight accuracy was examined with 5,000 nonparametric bootstrap samples. Of the 136 possible edges, 39 had bootstrapped 95% confidence intervals that excluded zero (36 positive, 3 negative); all edges with |w| ≥ 0.094 had confidence intervals excluding zero, whereas most smaller edges did not, consistent with the density of the estimated network (61% of edges nonzero).

Figure S2. Bootstrapped 95% confidence intervals of all edge weights (5,000 nonparametric bootstrap samples). The red line denotes the sample edge weights.

**S3. Full Edge-Weight Matrix**

The table lists the regularized partial correlation (edge weight) of every node pair in the EBICglasso network (tuning parameter γ = 0.5; polychoric correlations via automatic correlation selection). Edges estimated as exactly zero are shown as 0.

**Table S3. Edge weights (regularized partial correlations) for all 136 node pairs.**

| **Node1** | **Node2** | **Weight** |
| --- | --- | --- |
| ANX1 | ANX2 | 0.3177 |
| ANX1 | ANX3 | 0.1414 |
| ANX2 | ANX3 | 0.2456 |
| ANX1 | ANX4 | 0.1155 |
| ANX2 | ANX4 | 0.125 |
| ANX3 | ANX4 | 0.1883 |
| ANX1 | ANX5 | -0.0087 |
| ANX2 | ANX5 | 0 |
| ANX3 | ANX5 | -0.0075 |
| ANX4 | ANX5 | 0.1451 |
| ANX1 | ANX6 | 0.0493 |
| ANX2 | ANX6 | 0.1811 |
| ANX3 | ANX6 | 0.1384 |
| ANX4 | ANX6 | 0.1337 |
| ANX5 | ANX6 | 0.1231 |
| ANX1 | ANX7 | 0.0184 |
| ANX2 | ANX7 | 0.1285 |
| ANX3 | ANX7 | 0.1156 |
| ANX4 | ANX7 | 0.1251 |
| ANX5 | ANX7 | 0.0859 |
| ANX6 | ANX7 | 0.1132 |
| ANX1 | ANX8 | 0.1267 |
| ANX2 | ANX8 | 0.1442 |
| ANX3 | ANX8 | 0.0481 |
| ANX4 | ANX8 | 0.1901 |
| ANX5 | ANX8 | 0 |
| ANX6 | ANX8 | 0.3017 |
| ANX7 | ANX8 | 0.2823 |
| ANX1 | ENJ1 | 0 |
| ANX2 | ENJ1 | 0.0101 |
| ANX3 | ENJ1 | 0 |
| ANX4 | ENJ1 | 0 |
| ANX5 | ENJ1 | 0 |
| ANX6 | ENJ1 | 0 |
| ANX7 | ENJ1 | 0.0096 |
| ANX8 | ENJ1 | 0 |
| ANX1 | ENJ2 | 0.009 |
| ANX2 | ENJ2 | 0.0045 |
| ANX3 | ENJ2 | 0.0325 |
| ANX4 | ENJ2 | -0.0417 |
| ANX5 | ENJ2 | 0 |
| ANX6 | ENJ2 | 0 |
| ANX7 | ENJ2 | 0 |
| ANX8 | ENJ2 | 0 |
| ENJ1 | ENJ2 | 0.4376 |
| ANX1 | ENJ3 | 0 |
| ANX2 | ENJ3 | 0 |
| ANX3 | ENJ3 | 0.026 |
| ANX4 | ENJ3 | 0 |
| ANX5 | ENJ3 | -0.0215 |
| ANX6 | ENJ3 | 0 |
| ANX7 | ENJ3 | 0 |
| ANX8 | ENJ3 | 0 |
| ENJ1 | ENJ3 | 0.465 |
| ENJ2 | ENJ3 | 0.5407 |
| ANX1 | ENJ4 | 0 |
| ANX2 | ENJ4 | 0 |
| ANX3 | ENJ4 | -0.0648 |
| ANX4 | ENJ4 | 0.0093 |
| ANX5 | ENJ4 | 0.0649 |
| ANX6 | ENJ4 | -0.0102 |
| ANX7 | ENJ4 | 0 |
| ANX8 | ENJ4 | -0.0474 |
| ENJ1 | ENJ4 | 0.0939 |
| ENJ2 | ENJ4 | 0 |
| ENJ3 | ENJ4 | 0.0647 |
| ANX1 | ENJ5 | 0.0049 |
| ANX2 | ENJ5 | 0.0014 |
| ANX3 | ENJ5 | 0.0303 |
| ANX4 | ENJ5 | -0.0025 |
| ANX5 | ENJ5 | -0.0087 |
| ANX6 | ENJ5 | 0 |
| ANX7 | ENJ5 | 0.0001 |
| ANX8 | ENJ5 | 0 |
| ENJ1 | ENJ5 | 0.0102 |
| ENJ2 | ENJ5 | 0 |
| ENJ3 | ENJ5 | 0.0637 |
| ENJ4 | ENJ5 | 0.4432 |
| ANX1 | ENJ6 | -0.0457 |
| ANX2 | ENJ6 | -0.0398 |
| ANX3 | ENJ6 | -0.033 |
| ANX4 | ENJ6 | 0.0314 |
| ANX5 | ENJ6 | 0.1586 |
| ANX6 | ENJ6 | 0 |
| ANX7 | ENJ6 | -0.0524 |
| ANX8 | ENJ6 | 0 |
| ENJ1 | ENJ6 | -0.0028 |
| ENJ2 | ENJ6 | -0.0077 |
| ENJ3 | ENJ6 | -0.0155 |
| ENJ4 | ENJ6 | 0.1569 |
| ENJ5 | ENJ6 | 0.1072 |
| ANX1 | ENJ7 | 0.028 |
| ANX2 | ENJ7 | 0 |
| ANX3 | ENJ7 | -0.042 |
| ANX4 | ENJ7 | 0.0143 |
| ANX5 | ENJ7 | -0.0039 |
| ANX6 | ENJ7 | 0 |
| ANX7 | ENJ7 | 0 |
| ANX8 | ENJ7 | 0.0076 |
| ENJ1 | ENJ7 | 0 |
| ENJ2 | ENJ7 | 0 |
| ENJ3 | ENJ7 | 0.0085 |
| ENJ4 | ENJ7 | 0 |
| ENJ5 | ENJ7 | 0 |
| ENJ6 | ENJ7 | 0.25 |
| ANX1 | ENJ8 | 0 |
| ANX2 | ENJ8 | 0 |
| ANX3 | ENJ8 | -0.0192 |
| ANX4 | ENJ8 | 0.0134 |
| ANX5 | ENJ8 | 0 |
| ANX6 | ENJ8 | 0 |
| ANX7 | ENJ8 | 0.0064 |
| ANX8 | ENJ8 | 0 |
| ENJ1 | ENJ8 | 0 |
| ENJ2 | ENJ8 | 0 |
| ENJ3 | ENJ8 | 0 |
| ENJ4 | ENJ8 | 0.0074 |
| ENJ5 | ENJ8 | 0 |
| ENJ6 | ENJ8 | 0.1742 |
| ENJ7 | ENJ8 | 0.2544 |
| ANX1 | ENJ9 | 0 |
| ANX2 | ENJ9 | 0 |
| ANX3 | ENJ9 | -0.0187 |
| ANX4 | ENJ9 | 0 |
| ANX5 | ENJ9 | 0 |
| ANX6 | ENJ9 | 0 |
| ANX7 | ENJ9 | 0 |
| ANX8 | ENJ9 | 0 |
| ENJ1 | ENJ9 | 0.0169 |
| ENJ2 | ENJ9 | 0 |
| ENJ3 | ENJ9 | 0 |
| ENJ4 | ENJ9 | 0 |
| ENJ5 | ENJ9 | 0.2595 |
| ENJ6 | ENJ9 | 0.0511 |
| ENJ7 | ENJ9 | 0.2994 |
| ENJ8 | ENJ9 | 0.5873 |

**S4. Pairwise Difference Tests of KL Divergences（KL散度两两差异检验）**

Nonparametric bootstrap pairwise difference tests (5,000 resamples) were conducted on the KL divergences produced by manipulating each anxiety node at δ = 3, the smallest integer intensity at which at least one anxiety node reaches the effectiveness criterion in the anxiety→enjoyment direction (C = 3). (Note: the GGMNIRA package's global automatic intensity is C = 1 because it is defined across all construct directions of the joint network—including the reverse enjoyment→anxiety direction—which is not interpreted in this study.) Pairwise 95% confidence intervals were formed from the 2.5th and 97.5th percentiles of the resampled differences (Hyndman–Fan type 6, as implemented in differenceTest_KL()). Of the 28 unordered anxiety-node pairs (56 directed comparisons), 21 pairs (42 directed comparisons) differed significantly; self-comparisons are excluded. The top tier (ANX2, ANX8, ANX3) showed no significant pairwise differences among its members, whereas each top-tier node exceeded every middle-tier node (ANX1, ANX4, ANX6, ANX7); ANX5 fell significantly below all seven other nodes (all 95% CIs of the differences excluded zero). Because the bootstrap networks are re-estimated with Pearson correlations by package design, the absolute scale of the bootstrap KL values is not directly comparable to the sample KL values; conclusions rest on the ordering and the pairwise tests. Statistical power of these tests is limited at the present sample size (well below the n = 5,000 at which the framework's simulation study reached high power; 19), so non-significant pairs are described as “not significantly different” rather than “equal”. No multiplicity correction was applied, following the framework's recommendation.

Figure S4. Pairwise difference tests among node-manipulation KL divergences. Dark cells denote significant differences (95% bootstrap CI of the difference excludes zero).

**S5. Additional Difference Tests: EI, BEI, and Edge Weights**

Nonparametric bootstrap difference tests (5,000 resamples) among node centralities and edge weights: 90 of 136 pairwise EI comparisons (66.2%) and 52 of 136 pairwise BEI comparisons (38.2%) were significant; among the 83 nonzero edges, 2,050 of 3,403 pairwise edge-weight comparisons (60.2%) were significant. Dark cells denote significant differences.

Figure S5a. Pairwise difference tests of expected influence.

Figure S5b. Pairwise difference tests of bridge expected influence.

Figure S5c. Pairwise difference tests of edge weights.
